# Association Between Developmental Coordination Disorder Traits, Autistic Traits, and Emotional/Behavioral Problems in Japanese Preschool Children

**DOI:** 10.3390/children12040420

**Published:** 2025-03-27

**Authors:** Sumika Fujisawa, Aya Saito, Masumi Sugawara, Akio Nakai

**Affiliations:** 1 Graduate School of Humanities and Sciences, Ochanomizu University, Tokyo 112-8610, Japan; 2 Faculty of Core Research Human Science Division, Ochanomizu University, Tokyo 112-8610, Japan; 3 Department of Developmental Psychology, Faculty of Human Studies, Shirayuri University, Tokyo 182-8525, Japan; 4 Research Institute for Education & Graduate School of Clinical Education, Mukogawa Women’s University, Nishinomiya 663-8558, Japan

**Keywords:** developmental coordination disorder, autistic traits, emotional/behavioral problems, preschool children, developmental coordination disorder questionnaire–Japanese version (DCDQ-J)

## Abstract

**Background/Objectives:** Few studies have examined the association between developmental coordination disorder (DCD) traits and emotional/behavioral problems in preschool children, considering the influence of autistic traits. Furthermore, no consistent results have been obtained. The aim of this study was to investigate how DCD traits in preschool children are related to emotional/behavioral problems, controlling for the effects of autistic traits. **Methods:** A questionnaire survey was administered to 277 parents of children (154 boys, 73.0 ± 3.8 months) who underwent the school physical examination in Y city near Tokyo, Japan, from October to December 2021. The Developmental Coordination Disorder Questionnaire–Japanese version (DCDQ-J) was used to measure DCD traits, the Autism Spectrum Screening Questionnaire (ASSQ) was used to measure autistic traits, and the Strengths and Difficulties Questionnaire (SDQ) was used to measure emotional/behavioral problems. In the hierarchical multiple regression analysis, the SDQ was the dependent variable, with gender entered in Step 1, ASSQ in Step 2, and DCDQ in Step 3. **Results:** The results showed that autistic traits are associated with preschool children’s emotional/behavioral problems, but even after controlling for autistic traits, higher DCD traits had a relationship with higher conduct problems, hyperactivity/inattention, and peer problems and lower prosocial behavior of preschool children. **Conclusions:** This result indicates the need for the support of children with motor skill difficulties. Additionally, a focus on mitigating DCD traits not only improves motor skills but also prevents emotional/behavioral problems in preschool children.

## 1. Introduction

### 1.1. Developmental Coordination Disorder

Developmental coordination disorder (DCD) is a neurodevelopmental disorder categorized as a motor disorder in the *Diagnostic and Statistical Manual of Mental Disorders*, Fifth Edition, Text Revision (DSM-5-TR) [1]. DCD refers to a condition in which the acquisition and performance of coordinated motor skills are markedly lower than what would be expected given the person’s age and opportunities to acquire and use the skills. Moreover, the acquisition and performance are clearly inferior to those of other people, and as a result, they interfere with activities of daily life [1]. According to DSM-5-TR [1], the prevalence of DCD in school-aged children is 5–6%, and the most commonly cited rates in the literature range from 2% to 20%. The prevalence of DCD is higher in men than in women, with sex ratios varying from 2:1 to 7:1, depending on the study [1].

As children with DCD grow, more significant challenges arise as the demands placed on them increase [2]. These challenges include lower self-esteem, increased social isolation [3], increased peer relationship problems, and decreased social participation [4,5].

Motor coordination difficulties have been shown to be associated with difficulties in writing and learning, including learning mathematics, and cognitive skills, such as completing tasks that require simultaneous processing [6,7,8]. Moreover, longitudinal studies have found that difficulties with motor coordination from preschool onward lead to long-term school maladjustment, such as low academic performance, peer problems, and emotional symptoms, and conduct problems after school [9].

Additionally, it is estimated that 70% of children with DCD will continue to have coordination problems into adolescence and adulthood [1,10]. These include difficulties in learning various motor and new skills, such as driving [11]. The effects of DCD extend further than motor skills alone [12]. Adults with DCD traits experience lower social participation, lower life satisfaction, anxiety disorders and depression, obesity, cardiovascular disorders from lifestyle-related diseases, osteoporosis, and fractures [11,12,13,14,15]. These decrease life expectancy and healthy life expectancy; therefore, early recognition and appropriate support are necessary.

However, DCD traits are often overlooked as a developmental problem [16] and are under-recognized by caregivers, teachers, allied health professionals, medical professionals, psychotherapists, and clinical psychologists [17,18]. Furthermore, in Japan, the recognition that clumsiness is caused by a developmental problem in the brain function of coordination is lacking, and appropriate support has been delayed [19].

### 1.2. Association Between DCD Traits and Preschool Children’s Emotional/Behavioral Problems

DCD traits in preschool children are directly associated with behavioral/emotional problems [19,20] and influence internalizing problems through peer relationship problems and social skills [21,22]. Moreover, DCD affects play, which is central to young children’s lives. Children with DCD are more involved in aggressive behavior (both as a victim and perpetrator) in free play situations [23] and, compared with children without DCD, engage in less diverse, social, and frequent play [24]. Thus, DCD traits are a problem of poor motor skills and further develop into secondary problems, such as emotional/behavioral problems.

Research on the relationship between DCD traits and emotional/behavioral problems has been less extensive for preschool children compared to school-aged children [19,25,26]. Additionally, how coordination problems manifest as behavioral/emotional problems may differ depending on the age of the child, especially before and after school [19], suggesting that further research is needed. Furthermore, DCD is highly comorbid with other developmental disorders, which highlights the need to include the association with other developmental disorders in the research [26].

### 1.3. Association Between DCD and ASD Traits and Emotional/Behavioral Problems

The *Diagnostic and Statistical Manual of Mental Disorders*, Fourth Edition [27], did not permit a dual diagnosis of DCD with autistic spectrum disorder (ASD); however, the DSM-5 now permits this co-occurrence [11]. DCD has a high comorbidity rate with other developmental disorders, and approximately 80% of children with autistic traits are said to have difficulties with motor skills [28]. In Japan, one urban population-based study found that 63.2% of children with ASD have comorbid DCD [29].

Only a few studies have examined the relationship between DCD traits and children’s emotional/behavioral problems and included the influence of autistic traits. Studies with preschool children have found that compared with children without DCD, preschool children with a high likelihood of DCD were at a higher risk of scoring above clinical thresholds on each domain of the Strengths and Difficulties Questionnaire (SDQ) [30], a measure of emotional/behavioral problems. However, the association between DCD traits and emotional/behavioral problems became less significant when autistic traits were included in the analysis [26].

Furthermore, studies targeting school-aged children have indicated that children with DCD who also have comorbid autistic traits have a higher probability of having emotional/behavioral problems as they move up through the grades [31]. Thus, research on the coexistence of DCD traits and autistic traits in preschool children and its effects has not yet been sufficiently explanatory. Moreover, results consistent with studies conducted with school-aged children have not been obtained. Finally, although higher autistic traits in preschool children are associated with increased emotional/behavioral problems [29], it is not clear to what extent DCD traits alone are associated with emotional/behavioral problems when DCD and autistic traits coexist.

### 1.4. Purpose

The aim of this study was to investigate how DCD traits in preschool children are related to emotional/behavioral problems after controlling for the effects of autistic traits.

First, as gender differences have been reported in the scales used in this study, this study examined gender differences in each scale. Existing studies have reported that the Developmental Coordination Disorder Questionnaire ’07 (DCDQ), used as a measure of DCD traits, has been found to be scored higher by boys than by girls [32]. Similarly, boys have been found to score higher than girls on the Autism Spectrum Screening Questionnaire (ASSQ), which is used as a measure of autistic traits [33]. As for parents completing the SDQ, a scale used to measure emotional/ behavioral problems in children, it has been shown that boys score higher than girls in “conduct problems”, “hyperactivity/inattention”, and “peer problems”, and girls score higher than boys in “emotional symptoms” and “prosocial behavior” [34,35]. Based on these, it was assumed that gender differences would also be observed in this study. It was hypothesized that DCD traits would be associated with emotional/behavioral problems even after controlling for the effects of autistic traits. Although Shirama et al. [26] included children above the DCD cutoff, the present study treated DCD traits as a continuum. It targeted the general population to help capture the fine-scale association between DCD traits and emotional/behavioral problems.

## 2. Materials and Methods

### 2.1. Study Design

This was a multicenter cross-sectional study, performed between October and December of 2021, in 18 elementary schools in Y city near Tokyo, Japan.

### 2.2. Participants and Procedures

A set of questionnaires (informed consent and survey questionnaire) was distributed to parents at the school physical examination of 5- to 6-year-old children who were scheduled to enter elementary school the following year. Participants were asked to read the informed consent form and, if they agreed to participate in the questionnaire survey, to answer the questionnaire and return it by mail. With the cooperation of 18 out of 19 elementary schools in the city, the handouts were distributed to the parents of approximately 1550 children. Responses were obtained from 278 parents. Excluding one person whose answer was incomplete, 277 people were included in the analysis. The respondents were 261 mothers (94.2%) and 16 fathers (5.8%). The subjects were 154 boys (55.6%), 122 girls (44.0%), and 1 unknown (0.4%). The average age was 73.0 months (SD = 3.75).

### 2.3. Measures

#### 2.3.1. Developmental Coordination Disorder Traits

In this study, parents or caregivers completed the Japanese version of the DCDQ [32] developed by Willson et al. [36] to assess coordinative motor difficulties in children. This standard Japanese version of the DCDQ-J has been found to have good psychometric properties [32]. The DCDQ is the internationally recommended and most frequently used assessment tool for screening children with DCD [10,37]. The DCDQ consists of 15 items and 3 subscales, “Control During Movement” (6 items), “Fine Motor/Handwriting” (4 items), and “General Coordination” (4 items). The possible score range is 15 to 75 points; the items are scored on a 5-point system of 1 to 5. Lower total scores indicate greater difficulty with motor coordination. The total Cronbach’s alpha coefficient was 0.89.

#### 2.3.2. Autistic Traits

In this study, parents completed the Japanese version of the ASSQ short form to assess their children’s autistic traits [33]. The original Japanese version of the ASSQ [38] consists of 27 items that ask whether the traits of sociality, language, behavior, and interests typically seen in autism apply to the target person. In this study, we used the ASSQ short form, which, from its original version, had been narrowed down to 11 items with three factors with unique predictive power for identifying ASD, and each factor had a high γ. Equivalent to the original version, the ASSQ short form shows sufficient internal consistency and high discrimination for the diagnosis of ASD. The ASSQ short form is a screening tool for school-age children, but in Japan, it has been verified that the ASSQ shortened version can also be applied to preschool children [39,40]. The ASSQ short form consists of a three-factor structure of “unique interest” (three items), “sociality” (four items), and “friendship” (four items). The possible score range is 0 to 22 points; each item is rated using a 3-point system of 0 to 2 points. Higher scores indicate more pronounced autistic traits. The total Cronbach’s alpha coefficient was 0.76.

#### 2.3.3. Emotional and Behavioral Problems

In this study, parents completed the SDQ [30], a child behavior screening tool that has been shown to help identify the need for support for emotional/behavior problems to assess emotional/behavioral problems in their children. The SDQ is composed of 5 subscales, consisting of 4 difficulties, “emotional symptoms”, “conduct problems”, “hyperactivity/inattention”, and “peer problems”, and 1 strength: “prosocial behavior”. Each subscale consists of 5 items, for a total of 25 items. The possible score range for each subscale is 0–10 points; each item is rated using a 3-point system of 0–2 points. Higher scores indicate greater difficulty (except for “prosocial behavior”). Previous studies have validated the Japanese version of the SDQ [34,35]. Cronbach’s alpha coefficients for the subscales ranged from 0.46 (conduct problems) to 0.74 (prosocial behavior).

### 2.4. Ethical Considerations

This study was approved by the Ethics Committee of Ochanomizu University, Japan (approval no. 2021-132). Participants read the informed consent form and indicated their agreement to participate in the study by submitting the completed questionnaire.

### 2.5. Data Analyses

First, we conducted an independent t-test to examine whether there were gender differences in the SDQ, the ASSQ, and the DCDQ scores. Next, to examine the relationship between DCD traits and emotional/behavior problems, we conducted a hierarchical multiple regression analysis with the SDQ as the dependent variable. Note that the forced entry methods were used in this study as all independent variables were important for testing the hypotheses. Because gender differences were observed in some of the scales used in the previous t-test, gender was included in Step 1. To clarify the relationship between DCD traits and emotional/behavioral problems after considering the influence of autistic traits, first, in Step 2, only the ASSQ was input before DCD traits. In Step 3, the DCDQ was added as an independent variable to confirm whether DCD traits continued to show a unique and significant relationship with emotional/behavioral problems even after accounting for the effects of gender and autistic traits. All statistical analyses were conducted with IBM SPSS Statistics, version 26 (IBM Corporation, Armonk, NY, USA).

## 3. Results

### 3.1. Descriptive Statistics

Table 1 shows the means (SD) of the SDQ subscales, the ASSQ total, the DCDQ total, and the comparison between boys and girls. An independent t-test was used to examine the difference in scores between girls and boys. Note that for scales where the magnitude of variance was not considered equal (the ASSQ total and “hyperactivity/inattention”, “peer problems”, and “prosocial behavior” on the SDQ), *t*-tests using Welch’s method were conducted. The items of the SDQ on which boys scored significantly higher than girls were “hyperactivity/inattention” (*t*(270) = 2.41, *p* < 0.05) and “peer problems” (*t*(270) = 2.12, *p* < 0.05). They also scored higher on the ASSQ (*t*(270) = 4.06, *p* < 0.001). Girls scored significantly higher than boys on “prosocial behavior” (*t*(271) = −2.16, *p* < 0.05) on the SDQ. There were no significant differences between boys and girls on the SDQ “emotional symptoms” (*t*(273) = 0.87, *p* = 0.39) and “conduct problems” (*t*(271) = 1.50, *p* = 0.14) and the DCDQ (*t*(263) = 0.58 *p* = 0.56).

### 3.2. Hierarchical Multiple Regression Analysis

To investigate the relationship between DCD traits and children’s emotional/behavior problems, we performed hierarchical multiple regression analysis with the SDQ as the dependent variable and gender, autistic traits, and DCD traits as independent variables (Table 2). The presence or absence of multicollinearity was confirmed using the variance inflation factor statistic. The variance inflation factors of all predictors were less than 1.17, indicating that multicollinearity was not a problem in this study. The residue analysis showed that the Durbin–Watson scores ranged from 1.80 to 2.23, generally close to 2, which was not a problem in this study. There were also five people (1.8% of all cases) with an SD greater than 3 SD. This rate approximates the prevalence of ASD in the general population [1]. As the present study was aimed at the general community, the five people above 3 SD were included in the analysis.

Here, as gender differences were found in some of the SDQ subscales and ASSQ scores, gender was entered in Step 1, ASSQ was entered in Step 2, and DCDQ was entered in Step 3. All models were significant. In Step 2, it was clarified that autistic traits were significantly associated with children’s emotional/behavioral problems. Furthermore, from Step 2 to Step 3, dependent variables other than “emotional symptoms” showed a significant association, and the amount of change in the coefficient of determination increased significantly. In the dependent variables other than “emotional symptoms”, it became clear that the higher the DCD traits were, the higher “conduct problems”, “hyperactivity/inattention”, and “peer problems” were and the lower “prosocial behavior” was.

## 4. Discussion

The results of this study show that even after controlling for autistic traits, DCD traits have a significant relationship with preschool children’s higher “conduct problems”, “hyperactivity/inattention”, and “peer problems” and lower “prosocial behavior”. Thus, DCD traits were found to be associated with emotional/behavior problems, except for one subscale of the SDQ (“emotional symptoms”), even after controlling for autistic traits.

Many previous studies have reported the association between DCD traits and internalizing problems in preschool children [21,22]. However, this study also confirmed a relationship with externalizing problems, such as “conduct problems” and “hyperactivity/inattention”.

The finding of an association between DCD traits and “hyperactivity/inattention” is consistent with those of many previous studies [31,41]. Although the comorbidity of attention deficit hyperactivity disorder and DCD is high at approximately 50% [1], problems of “hyperactivity/inattention” derived from DCD traits, such as standing and walking due to the immaturity of motor skills, have also been noted [19]. Therefore, given the perspective of DCD traits as one of the factors related to “hyperactivity/inattention” in children, it is important to provide support for children with DCD traits.

The results of the present study, in which DCD traits were associated with higher “peer problems” and “conduct problems” and lower “prosocial behavior”, are in line with previous research showing that children with DCD are more involved in aggressive behavior (both as victims and perpetrators) in free play situations [23] and engage in less diverse, social, and frequent play than children without DCD [24]. DCD traits result in reduced social participation [5], and aggressive behaviors may be associated with peer relationships and adaptation problems within the same age group. This suggests the need for appropriate and early interventions to address aggressive behaviors associated with DCD traits.

Early childhood is considered a period of learning through play, so children engage in activities that require fine motor skills, such as origami, drawing, and arts and crafts, as well as gross motor activities, such as playing with toys and using their bodies outside. Therefore, as shown in a previous study [24], immaturity in motor skills increases the likelihood that children will experience difficulties during play and activities of daily living that are central to their lives. Additionally, DCD traits may be associated with a wide range of emotional/behavioral problems, as shown in this study.

Conversely, in the hierarchical multiple regression analysis in the present study, no association with “emotional symptoms” was found after controlling for autistic traits. This result is consistent with that of Mikami et al. [17], who found no association with “emotional symptoms” in their study with preschool children. In another study with preschool children [21], no direct association of DCD traits and “emotional symptoms” was found, but DCD traits mediated the social skills and were associated with “emotional symptoms”. This suggests that the association between DCD traits and “emotional symptoms” in preschool children may emerge as a secondary problem mediated by another factor. In addition, given that a study with school-aged children [41] found an association between DCD traits and “emotional symptoms”, this direct association may emerge more prominently during schooling when more complex and advanced coordination is required [42].

In this study, a direct relationship was found between DCD traits and emotional/behavioral problems. Furthermore, previous research has shown that children’s motor skills are directly related to emotional/behavioral problems [19]. However, research has also shown that children’s motor skills are not directly related to emotional/behavioral problems but that peer problems and social skills mediate this relationship [21,22]. These differences may be due to using different scales to measure DCD traits. In the studies with a direct relationship, DCD traits were measured using the DCDQ, a parent-completed questionnaire.

In contrast, DCD traits with no direct relationship were measured with the Bruininks–Oseretsky Test of Motor Proficiency, Second Edition—short form (BOT-2SF), a professional direct motor skill rating. In other words, the DCDQ asks the extent to which a child demonstrates difficulties with motor skills in real life, whereas the BOT-2 directly measures difficulties with motor skills. Therefore, it can be inferred that compared with the direct level of motor skills, the extent to which motor skill difficulties manifest in real life may be more strongly related to children’s emotional/behavioral problems. In the study where no direct association was found, the mean age was 5:4–5:5 [21,22], whereas in the present study, the mean age was 6:0. This value was about six months older than in the previous study, although the same infants were included in the study. Despite this small difference, the results may support previous research showing that children with DCD traits experience various difficulties as they get older [2,3,4,5]. It may be necessary to examine the effects of grade and age in the same early childhood age group as it is suggested that the effects of age may be more significant in the early childhood age group than in the school-age group.

Additionally, this study found that DCD traits are associated with emotional/behavioral problems even after controlling for autistic traits. This result partly differs from previous research, which found that although associations with emotional/behavior problems were higher among preschool children with a high likelihood of DCD, these associations became less significant when autistic traits were included in the analysis, and only children with high autistic traits and a high likelihood of DCD had more negative emotional/behavior problems [26]. In the present study, DCD traits were associated with behavior problems regardless of the level of autistic traits; these differences may be influenced by the differences in the subject population. Moreover, they may occur due to the fact that the study by Shirama et al. [24] limited its analysis to children with DCD trait scores above a cutoff value. By contrast, the present study treated DCD traits as a continuum and included the entire sample of the general population. Further validation with a larger sample, including clinical groups with DCD, will be necessary.

It has also been suggested that reduced physical performance due to DCD traits may reduce participation in various activities and limit opportunities to improve physical performance. This reluctance to participate may lead to a negative feedback loop of reduced self-perception and self-efficacy, reducing motivation to participate in physical activity [43]. Interventions are needed for children with possible DCD, with or without a diagnosis [11], and interventions may be important to ensure that motor skill immaturity due to these DCD traits does not affect emotional/behavioral problems and lead to a negative loop. There are two main types of approaches for DCD: the task-oriented approach (analyzing motor performance during activities and the factors and context that influence it to help develop better strategies) and the process-based approach (working on the elements and bodily functions necessary for performing an activity) [14,32]. Of the two, the task-oriented approach is considered more effective as a support method for children with DCD traits because it targets activities essential to their daily life and allows families to encourage children’s participation in school activities, leisure time, and sports [11,16]. Cognitive orientation to daily occupational activities improved occupational performance and motor skills in an intervention study of children with DCD in Japan [44].

Despite the development of such support methods, 80% of children with autistic traits have problems with motor skills [28], while clumsiness is often due to problems with brain function. Because there is little recognition that this is happening [19], it seems that the immaturity of motor skills and the need for the support of children with autistic traits are easily overlooked. Insufficient support for DCD traits has been reported in the USA [45], Australia [46], and Canada [47] and is likely to be similar in Japan. Based on the results of this study, which shows that DCD traits are associated with children’s emotional/behavioral problems even after controlling for autistic traits, support for children with DCD traits should focus on not only improving their motor skills but also addressing secondary problems, such as aggressive behavior and peer problems that stem from poor motor skills. Therefore, it is important to incorporate the perspective of focusing on DCD traits into child development support.

The present study had several limitations. First, the small sample size may limit generalization. Therefore, it is desirable to conduct studies with larger sample sizes in the future. Second, the findings of this study on DCD traits, autistic traits, and emotional/behavioral problems were obtained from parental reports, and the possibility of being influenced by the evaluator cannot be ruled out. In particular, the items regarding emotional/behavioral problems may have included evaluations based on estimates of group behavior that parents were not fully aware of. A study examining whether parents and teachers differ in their ratings of the SDQ scores for children with DCD found that parents rated hyperactivity and prosocial behavior higher than teachers, pointing to the importance of obtaining ratings from both teachers and parents [48]. In the future, we would like to include objective indicators, such as evaluations by childcare workers and parents. Third, information on SES and other comorbid disorders was not included. In the future, other information lacking in this study should be included and discussed. Fourth, given that this study was a cross-sectional study and we did not obtain information on whether the participants had been formally diagnosed with DCD or ASD, it is difficult to judge the relationship from a temporal order perspective. In the future, it will be desirable to conduct longitudinal studies to accurately understand the relationship and verify the effectiveness of interventions for DCD traits.

Despite these limitations, we believe that this study has great value as it targeted preschool children, with whom only a few studies have been conducted, and examined the coexistence of DCD and autistic traits. These findings helped to demonstrate the need for support for preschool children with high levels of DCD traits. In the future, we will conduct a longitudinal study to examine the changes in children’s emotional/behavioral problems as they move from early childhood, which focuses on play, to school, which focuses on learning (requiring writing skills). Furthermore, we would like to examine the effects of support on children with DCD traits.

## 5. Conclusions

In this study, we administered a questionnaire survey to the parents of preschool children and examined the relationship between DCD traits and children’s emotional/behavioral problems, including autistic traits in the analysis. The results showed that even after controlling for autistic traits, a relationship was found with higher conduct problems, hyperactivity/inattention, and peer problems and lower prosocial behavior.

Although DCD traits are highly comorbid with ASD traits, the result that DCD traits are associated with emotional/behavioral problems even after controlling for autistic traits suggests that DCD traits may be overlooked: the inability to respond to adult requests or to join in with others due to DCD traits may be seen simply as a communication difficulty. In supporting children with DCD, it is necessary to not only improve motor skills but also to provide support from a multifaceted perspective, such as preventing emotional and behavioral problems in children. Therefore, collaboration among multiple professions and facilities, including childcare, education, medical care, rehabilitation (e.g., occupational therapy, physical therapy, and speech therapy), and welfare, is important.

In the future, we would like to examine longitudinally what types of emotional/behavioral problems emerge as children with DCD traits grow up, including ASD traits and other neurodevelopmental disorder traits, and to examine the effect of support for children with DCD traits on emotional/behavioral problems.

## Figures and Tables

**Table 1:** Descriptive statistics for the mean (SD) of each factor and t-test for gender differences.

	Total		Boys		Girls		Gender Group Comparison	
	*M*	*SD*	*M*	*SD*	*M*	*SD*	*t*	*p*
SDQ								
Emotional symptoms	1.79	(1.95)	1.88	(1.87)	1.67	(2.06)	0.87	0.39
Conduct problems	1.79	(1.51)	1.92	(1.61)	1.64	(1.36)	1.50	0.14
Hyperactivity/inattention	2.96	(2.18)	3.24	(2.29)	2.62	(1.98)	2.41 *	<0.05
Peer problems	1.44	(1.54)	1.61	(1.74)	1.23	(1.24)	2.12 *	<0.05
Prosocial behavior	6.89	(2.20)	6.66	(2.45)	7.21	(1.80)	−2.16 *	<0.05
ASSQ	2.64	(3.05)	3.27	(3.27)	1.83	(2.56)	4.06 ***	<0.001
DCDQ	53.61	(10.89)	53.96	(11.18)	53.17	(10.59)	0.58	0.56

*M*: Mean; *SD*: Standard deviation; SDQ: Strengths and Difficulties Questionnaire; ASSQ: Autism Spectrum Screening Questionnaire; DCDQ: Developmental Coordination Disorder Questionnaire. * *p* < 0.05, *** *p* < 0.001.

**Table 2:** Hierarchical multiple regression analysis of gender, ASSQ, and DCDQ at emotional/behavioral problems.

	Emotional Symptoms				Conduct Problems				Hyperactivity/Inattention				Peer Problems				Prosocial Behavior			
	B	SE B	Β	∆*R*^2^	B	SE B	β	∆*R*^2^	B	SE B	β	∆*R*^2^	B	SE B	β	∆*R*^2^	B	SE B	β	∆*R*^2^
Step 1				0.01				0.01				0.02 *				0.01				0.02 *
gender	−0.27	0.24	−0.07		−0.30	0.19	−0.10		−0.62	0.27	−0.14		−0.34	0.19	−0.11		0.56	0.28	0.13	
Step 2				0.09 ***				0.06 ***				0.12 ***				0.32 ***				0.03 **
gender	0.02	0.24	0.01		−0.12	0.19	−0.04		−0.23	0.26	−0.05		0.09	0.16	0.03		0.38	0.28	0.08	
ASSQ	0.20	0.04	0.31 ***		0.12	0.03	0.25 ***		0.26	0.04	0.36 ***		0.28	0.03	0.58 ***		−0.13	0.04	−0.18 **	
Step 3				0.01				0.01 *				0.09 ***				0.05 ***				0.03 **
gender	−0.01	0.24	−0.00		−0.17	0.19	−0.05		−0.39	0.25	−0.09		0.01	0.16	0.00		0.47	0.28	0.10	
ASSQ	0.18	0.04	0.29 ***		0.10	0.03	0.20 **		0.19	0.04	0.27 ***		0.25	0.03	0.51 ***		−0.09	0.05	−0.13	
DCDQ	−0.01	0.01	−0.08		−0.02	0.01	−0.13 *		−0.06	0.01	−0.31 ***		−0.03	0.01	−0.24 ***		0.03	0.01	0.17 **	
*R* ^2^	0.10 ***				0.08 ***				0.23 ***				0.38 ***				0.07 ***			
adj *R*^2^	0.09 ***				0.07 ***				0.22 ***				0.38 ***				0.06 ***			

*R*^2^ and adj *R*^2^ are listed for Step 3. * *p* < 0.05, ** *p* < 0.01, *** *p* < 0.001. ASSQ: Autistic Spectrum Screening Questionnaire. DCDQ: Developmental Coordination Disorder Questionnaire.

## Data Availability

This study is ongoing and to ensure the originality and accuracy of the study, the raw data supporting the conclusions of this article will be made available by the authors upon request.

## Abbreviations

The following abbreviations are used in this manuscript:

DCD	Developmental coordination disorder
SDQ	Strengths and Difficulties Questionnaire
ASD	Autism spectrum disorder
ASSQ	Autism Spectrum Screening Questionnaire
DCDQ	Developmental Coordination Disorder Questionnaire
DCDQ-J	Developmental Coordination Disorder Questionnaire–Japanese version
